# Pediatric MOG antibody-positive encephalitis with normal brain magnetic resonance imaging: a new spectrum associated with MOG antibodies?

**DOI:** 10.3389/fneur.2025.1537538

**Published:** 2025-02-26

**Authors:** Yan Jiang, Ping Yuan, Xiaojie Song, Jiannan Ma, Siqi Hong, Xiujuan Li, Li Jiang

**Affiliations:** Department of Neurology Children’s Hospital of Chongqing Medical University, National Clinical Research Center for Child Health and Disorders, Ministry of Education Key Laboratory of Child Development and Disorders, Chongqing Key Laboratory of Child Neurodevelopment and Cognitive Disorders, Chongqing, China

**Keywords:** encephalitis, brain magnetic resonance imaging, myelin oligodendrocyte glycoprotein antibody, myelin oligodendrocyte glycoprotein antibody-associated disease, immunotherapy

## Abstract

**Objective:**

To facilitate the accurate identification of clinical characteristics associated with myelin oligodendrocyte glycoprotein (MOG) antibody positive encephalitis in children presenting with normal brain magnetic resonance imaging (MRI) findings.

**Method:**

Patients hospitalized at Children’s Hospital of Chongqing Medical University from January 2016 to May 2024, who were positive for MOG antibodies and exhibited encephalitis symptoms with normal brain MRI findings, were retrospectively analyzed.

**Results:**

A total of 17 patients (7 males and 10 females; mean age: 9.2 ± 2.8 years) were enrolled in the study. The most prevalent clinical symptoms were fever (17/17), with a median duration of 15 days (IQR: 7.5–21 days), headaches (17/17), mild alterations in mental status (17/17), seizures (6/17), vomiting (6/17), decreased binocular vision (2/17), and hemiplegia (1/17). The majority of cases (15/17) exhibited leukocytosis in peripheral blood (mean: 20.63 ± 7.09 × 10^9^/L) accompanied by an elevated neutrophil ratio. C-reactive protein (CRP) and procalcitonin (PCT) levels were normal in 13 patients (13/17). Cerebrospinal fluid (CSF) leukocyte counts were elevated in all patients (median: 82/mm^3^; IQR: 49–155/mm^3^). Six patients (6/17) had elevated CSF protein levels (mean: 1.01 ± 0.38 g/L). CSF glucose levels were normal across all patients. Next-generation sequencing of CSF was performed in 10 patients, all yielding negative results. All patients had a serum MOG antibodies titer of ≥1:32, and six children (6/17) had a CSF MOG antibody titer of ≥1:32. All patients showed clinical improvement after immunotherapy. Only one patient (1/17) experienced a relapse.

**Conclusion:**

For patients presenting with encephalitis and normal brain MRI findings, early testing for anti-MOG antibody should be considered if they exhibit the following characteristics: (1) persistent fever; (2) elevated peripheral blood white blood cell (WBC) counts, with normal or slightly elevated PCT and CRP levels; (3) mild elevation of CSF WBC counts, normal or mildly elevated protein levels, and normal CSF glucose levels; and (4) ineffectiveness of antibiotic or antiviral therapy. Encephalitis with normal brain MRI may be regarded as a potential new spectrum associated with MOG antibodies, meriting additional exploration and consideration.

## 1 Introduction

The typical phenotypes of myelin oligodendrocyte glycoprotein antibody-associated disease (MOGAD) in children include acute disseminated encephalomyelitis (ADEM), optic neuritis (ON), neuromyelitis optica spectrum disorder, and transverse myelitis. According to the criteria proposed by the International MOGAD Panel, the core demyelinating events are ON, myelitis, ADEM, cerebral monofocal or polyfocal deficits, brainstem or cerebellar deficits and cerebral cortical encephalitis often with seizures (1). Recently, the disease spectrum has expanded with clinical and radiological patterns, such as aseptic meningitis (2, 3), isolated seizures (4), and other non-classifiable presentations. Notably, in some atypical phenotypes, like isolated seizures and aseptic meningitis (3, 5), some cases exhibit normal brain MRI findings, contrasting with the typical presentation of MOGAD. Currently, sporadic cases of MOG antibody-positive encephalitis with normal brain MRI (3, 6–8) and some cases of MOG antibody-positive myelitis accompanied by a negative spinal cord MRI have been documented (9), which do not conform to the latest criteria proposed by the International MOGAD Panel. Our center has previously observed and reported this phenomenon, albeit in a limited number of cases (10, 11). MOG antibodies positive encephalitis with normal brain MRI can easily be misdiagnosed as intracranial infection, leading to delayed treatment. However, there is limited research on this topic.

Therefore, we present a report on 17 patients from our single center who have been diagnosed with MOG antibodies positive encephalitis, despite exhibiting normal brain MRI findings. This report aims to enhance clinicians’ understanding of this newly recognized phenotype, thereby facilitating earlier diagnosis and more timely treatment. To our knowledge, this is currently the largest case series of MOG antibodies positive pediatric autoimmune encephalitis with normal brain MRI to date. Initially, some patients at our center exhibited normal brain imaging results. As the disease advanced, subsequent brain imaging scans unveiled new lesions in these individuals. Ultimately, they were diagnosed with ADEM, cortical encephalitis, or cerebral monofocal/polyfocal deficits. This phenomenon has also been reported in previous literature (8, 12). However, these pediatric patients were excluded from this study.

## 2 Subject and method

We retrospectively analyzed patients aged 18 years or younger with serum positive MOG antibodies, proven by fixed or lived cell-based assays (FCBA or LCBA), hospitalized in the Department of Neurology of Children’s Hospital of Chongqing Medical University, China, from January 2016 to May 2024. We selected patients with encephalitis who had normal brain MRI without meningeal enhancement and intracranial lesions. Encephalitis was defined according to international criteria for encephalitis (13). Patients who progressed during the acute phase of the disease (3 months for ADEM and 1 month for other encephalitis) (6) and were ultimately diagnosed with ADEM, cortical encephalitis, monofocal/polyfocal deficits were excluded. Besides, patients whose vision declined later but had normal brain imaging were not excluded. We defined an acute episode as a new neurological deficit lasting at least 24 h. Relapses we redefined as new neurological symptoms 1 month after the onset or, in the case of ADEM, as two episodes separated by 3 months (14, 15). The modified Rankin scale (mRS) was used to assess the short-term prognosis of the children (6). mRS ≥ 2 at the last follow-up was considered a poor outcome.

The following detailed clinical information was collected: the demographic characteristics, symptoms, physical signs, complete blood count, serum C-reactive protein (CRP) level, erythrocyte sedimentation rate, serum procalcitonin (PCT) level, CSF examination, serum and/or cerebrospinal fluid MOG-IgG antibody titer, radiological features, electroencephalographs (EEG), treatment information and their outcomes.

The brain MRI scanning procedure was performed utilizing either a 1.5 Tesla (T) or 3.0 T machine, wherein images were obtained for the following sequences: T1-weighted imaging (T1WI), T2-weighted imaging (T2WI), T2 fluid-attenuated inversion recovery (FLAIR), contrast-enhanced T1-weighted imaging, diffusion-weighted imaging (DWI), and apparent diffusion coefficient (ADC) mapping.

Serum and CSF MOG antibodies were detected by fixed or lived cell-based assay (FCBA or LCBA). Serum and CSF anti-aquaporin-4 (AQP4) antibody and anti-glial fibrillar acidic protein (GFAP) antibodies were detected by FCBA. Antibody titers of ≥1:32 were considered positive.

All statistical analyses were performed with SPSS 25. Continuous variables were expressed as means ± standard deviation (SD) if normally distributed or as median and interquartile range (IQR) if nonnormally distributed. Count data were expressed as number of cases and percentage (%). This study was approved by the Ethics Committee of the Children’s Hospital of Chongqing Medical University. The informed consents were obtained from the patients’ parents.

## 3 Results

### 3.1 The clinical findings

We identified 17 patients (17/364, 4.67%) who presented with an encephalitis phenotype and normal brain MRI from a cohort of 364 patients diagnosed with MOGAD, including 7 males and 10 females. All participants had a history of good physical health and no prior neurological or systemic immune diseases. The detailed demographic and clinical features were summarized in Table 1. At the onset, the mean age of the patients was 9.2 ± 2.8 years. During the acute phase of encephalitis, all patients presented with fever, lasting for 15 days (IQR: 7.5–21 days). Other symptoms included headaches (17/17, 100%), slightly altered mental status (17/17, 100%); seizures (6/17, 35%), with one case involving focal convulsion status; vomiting (6/17, 35%); decreased binocular vision (2/17, 12%), occurring on the 20th and 28th day of the disease course, respectively; hemiplegia (1/17, 6%). The modified Rankin score (mRS) was 2 (IQR: 2–3).

**Table 1:** The detailed demographic and clinical features of the pediatric patients with MOG antibody-positive and normal brain MRI.

Patient	Sex	Age (y)	Symptoms	Duration of fever (days)	mRS at onset	Serum (WBC/N/CRP/PCT)	CSF (WBC/Pro/Glu/NGS)	MOG-Ab titer in serum/CSF	EEG
1	M	3.3	Fever, headache, lethargy, seizure	15	3	33.61/0.76/<8/<0.05	240/0.85/2.96/−	1:100/− (FCBA)	Generalized slowing
2	F	11.3	Fever, vomiting, headache, lethargy, seizure	5	3	13.20/0.75/<8/<0.05	75/0.33/4.45/NA	1:100/1:100 (FCBA)	Generalized slowing
3	F	9.7	Fever, vomiting, headache, lethargy	15	3	19.50/0.81/<8/<0.05	362/1.48/2.7/NA	1:32/1:10 (FCBA)	Generalized slowing
4	F	11.3	Fever, headache, lethargy, seizure, right hemiplegia	10	4	21.50/0.86/<8/<0.05	22/0.28/2.73/NA	1:32/1:10 (FCBA)	Generalized slowing, spike-slow wave in the left posterior temporal region
5	F	10.2	Fever, headaches, seizure, somnolence	13	2	10.83/0.76/<8/<0.05	28/0.36/2.63/NA	1:32/1:32 (FCBA)	Generalized slowing
6	F	10.6	Fever, headache, lethargy	21	2	12.35/0.83/<8/<0.05	111/0.37/2.9/−	1:100/1:10 (FCBA)	Normal
7	F	7.7	Fever, headache, vomiting, lethargy	21	2	24.81/0.83/<8/<0.05	216/0.67/2.33/−	1:32/− (FCBA)	Normal
8	F	10.4	Fever, vomiting, headache, lethargy, focal convulsion status	4	3	11.53/0.9/<8/0.07	41/0.37/2.89/−	−/− (FCBA) 1:100/1:32 (LCBA)	Slowing in the left temporal region
9	M	9.5	Fever, headache, lethargy	20	2	22.85/0.81/<8/<0.05	124/0.43/3.44/−	1:100/1:32 (FCBA) 1:100/1:32 (LCBA)	Normal
10	M	7.1	Fever, headache, lethargy	18	2	22.03/0.82/10.61/<0.05	82/0.23/3.14/−	1:100/− (FCBA)	Normal
11	F	4.9	Fever, headache, lethargy	5	2	22.39/0.87/36.62/0.07	80/0.24/2.82/NA	1:100/1:10 (FCBA)	Normal
12	M	7.8	Fever, headache, lethargy, seizure	10	2	29.70/0.83/54.18/0.14	46/0.55/3.29/NA	1:32/1:10 (FBCA) 1:100/− (LBCA)	Generalized slowing
13	M	7.9	Fever, headache, lethargy,	3	2	26.81/0.75/20.97/<0.05	57/0.28/2.7/−	1:320/− (FCBA)	Generalized slowing
14	M	14.5	Fever, vomiting, headache, lethargy	24	2	17.55/0.84/<8/<0.05	110/1.4/2.61/−	1:32/1:32 (FCBA)	Normal
15	F	7.1	Fever, vomiting, headache, lethargy, decreased binocular vision	23	3	17.47/0.84/<8/<0.05	52/0.18/2.77/−	1:32/− (FCBA)	Generalized slowing
16	M	13.5	Fever, headache, lethargy, decreased binocular vision	30	3	13.40/0.75/<8/<0.05	185/0.38/2.55/−	−/− (FCBA) 1:100/− (LCBA)	Slowing in the bilateral frontal region
17	F	10.1	Fever, headache, lethargy	21	2	31.23/0.89/<8/0.09	126/1.11/2.52/NA	1:32/1:32 (FCBA)	Normal

M, male; F, female; y, years; mRS, modified Rankin scale; N−, negative; NA, not applicable; m, months; serum WBC, peripheral white blood cell count (×10^9^/L); N, neutrophil ratio; CRP, C-reactive protein (mg/L); PCT, procalcitonin (ng/m); Pro, CSF total protein (mg/dl); CSF WBC, white blood cells/mm^3^ in CSF; Glu, CSF glucose (mmol/L); NGS: next-generation sequencing; FCBA: fixed cell-based assay; LCBA: lived cell-based assay; EEG, electroencephalography.

### 3.2 The ancillary examination results

The average blood white blood cell (WBC) count was 20.63 ± 7.09 × 10^9^/L, with the majority of cases (15/17, 88%) exhibiting leukocytosis and elevated neutrophil ratios in all patients. However, C-reaction protein (CRP) and procalcitonin (PCT) were normal in 13 patients (13/17, 76%). Four of seventeen (24%) patients had elevated CRP (31.60 ± 19.01 mg/L, normal value: <8 mg/L). PCT were mildly elevated in four patients (24%) (range: 0.07–0.14 ng/mL, normal value: <0.05 ng/mL). CSF leukocytes were elevated in all patients (median: 82/mm^3^, IQR: 49–155/mm^3^). CSF protein was elevated in six patients (6/17, 35%) (mean: 1.01 ± 0.38 g/L, normal value: ≤0.45 g/L). CSF glucose levels were normal in all patients. Herpes simplex virus, Epstein–Barr virus, *Mycoplasma pneumoniae* and bacteria in the CSF were all negative. Ten patients (10/17, 59%) underwent next-generation sequencing testing of CSF, with all tests yielding negative results.

### 3.3 MOG antibodies in serum/CSF

All 17 children had serum MOG antibodies titers of ≥1:32. Thirteen patients underwent only FCBA testing, while four patients received both FCBA and LCBA testing. Patients 8 and 16 were negative by FCBA but had a titer of 1:100 by LCBA. MOG antibodies titers in CSF were ≥1:32 in six children (6/17, 35%), 1:10 in five children (5/17, 29%) and negative in six children (6/17, 35%). All patients tested negative for anti-AQP4 and anti-GFAP antibodies in both serum and CSF.

### 3.4 EEG findings

EEG were abnormal in 10 of the 17 children (10/17, 59%) during the acute phase, revealing generalized slowing in seven children (7/17, 41%), focal slow waves in two children (2/17, 12%), and generalized slowing combined with focal epileptic discharges in one child (1/17, 6%). The other seven children (7/17, 41%) had no significant EEG abnormalities.

### 3.5 Neuro-imaging

**Figure 1:**
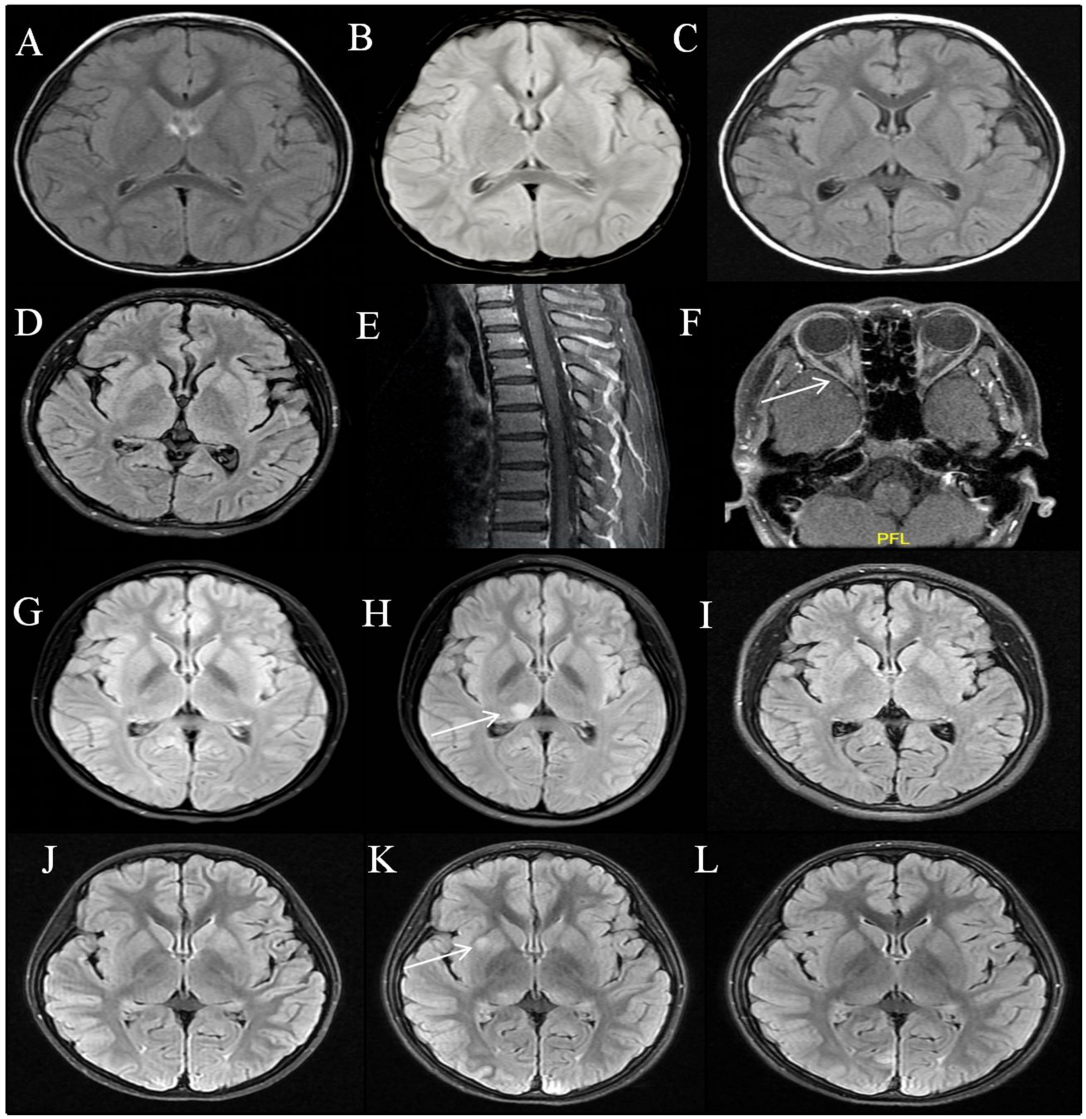
MRI features of MOG antibodies positive encephalitis. Patient 1: Normal brain MRI on the 11th, 20th, and 54th days from onset **(A–C)**; Patient 16: Normal brain and spinal MRI at acute phase **(D,E)**; Bilateral optic nerve enhancement on the 28th day after the onset **(F)**. Patient 2: Normal brain MRI at the first attack **(G)**; Six months after onset, a relapse occurred with T2 flair hyperintensity in the right thalamus **(H)**; The lesion disappeared after immunotherapy **(I)**. Patient 3: Normal brain MRI at acute phase **(J)**; T2 flair hyperintensity in the right globus pallidus on follow-up scans 2 months later **(K)**; The lesion almost disappeared 5 months after the onset of the disease without chronic immunotherapy **(L)**.

During the acute phase, 17 children underwent brain MRI scans which revealed normal results, with a median duration of 9 days (IQR: 5.5–16.5 days). Of the 17 patients, 15 underwent a follow-up brain MRI during the acute phase, with a median duration of 13 days (IQR: 9–23 days). None of the patients exhibited new intracranial lesions or meningeal enhancement (Figures 1A–C). Spinal MRI was also conducted in six patients with normal results. When patient 15 and 16 clinical manifestations of optic neuritis, orbital MRI revealed optic nerve inflammation (Figures 1D–F).

### 3.6 The treatments and outcomes

The treatments and outcomes of the 17 patients were summarized in Table 2. All patients were suspected of having an intracranial infection and received treatment with acyclovir or/and antibiotics before MOGAD diagnosis. However, there was no improvement in clinical outcomes, and two cases experienced visual impairment during the course of the disease. The median time to diagnosis after onset was 18 days (IQR: 10–25.5 days). Thirteen patients (13/17, 76%) were treated with intravenous immunoglobulin (IVIG) at 2 g/kg over 4–5 days after suspected or diagnosed MOGAD. Ten patients (10/17, 59%) were treated with IVIG combined with intravenous methylprednisolone (IVMP) at a dose of 15–20 mg/kg per day over 3–5 days in the acute phase following oral prednisone (OP, 1.5–2.0 mg/kg/day). Two patients (2/17, 12%) were treated with IVMP following OP, and one patient (1/17, 6%) was treated with IVIG combined with OP. Two patients (2/17, 12%) were treated with IVIG only, and two patients (2/17, 12%) was treated with OP alone. For the children who received OP, the dosage was gradually reduced and discontinued within a period of 3 to 6 months. All patients showed clinical improvement after immunotherapy treatment.

**Table 2:** The treatments and outcomes of the pediatric patients with MOG antibody-positive and normal brain MRI.

Patient	Initial diagnosis	Treatment before MOGAD diagnosis	Time to diagnosis (days)	Immunotherapy	Time to 2nd relapse (months)	Follow up (months)	mRS at last follow-up
1	Purulent meningitis	Vancomycin + meropenem	28	IVMP, OP	None	40	0
2	Viral encephalitis	Acyclovir	9	IVIG, IVMP, OP	Encephalitis/6	39	0
3	Purulent meningitis	Vancomycin + meropenem	14	IVIG	None	38	0
4	Viral encephalitis	Acyclovir, oxcarbazepine	10	IVIG, IVMP, OP	None	40	0
5	Purulent meningitis	Cephalosporin	70	OP	None	48	0
6	Viral encephalitis; Purulent meningitis	Cephalosporin + acyclovir	22	IVIG, IVMP, OP	None	12	0
7	Purulent meningitis	Cephalosporin	18	IVIG, IVMP, OP	None	8	0
8	Viral encephalitis	Acyclovir	7	OP	None	3	0
9	Viral encephalitis; Purulent meningitis	Cephalosporin + acyclovir	17	IVIG, IVMP, OP	None	4	0
10	Purulent meningitis	Vancomycin + meropenem	19	IVIG, OP	None	3	0
11	Purulent meningitis	Vancomycin + meropenem	10	IVIG, IVMP, OP	None	9	0
12	*Mycoplasma pneumoniae* encephalitis	Doxycycline	7	IVIG, IVMP, OP	None	9	0
13	Viral encephalitis; Purulent meningitis	Cephalosporin + acyclovir	10	IVIG, IVMP, OP	None	17	0
14	Purulent meningitis	Vancomycin + meropenem	20	IVIG	None	26	0
15	Viral encephalitis; Purulent meningitis	Cephalosporin + acyclovir	25	IVIG, IVMP, OP	None	42	0
16	Viral encephalitis	Acyclovir	30	IVIG, IVMP, OP	None	5	0
17	Purulent meningitis	Cephalosporin + vancomycin	26	IVMP, OP	None	40	0

IVIG, intravenous immunoglobulin; IVMP, intravenous methylprednisolone; OP, oral prednisone; mRS, modified Rankin scale.

All children were followed for a median duration of 17 months (IQR: 6.5–40 months). One child (1/17, 6%, patient 2) developed a relapse. Six months after the initial disease onset, patient 2 experienced a relapse with clinical manifestations of encephalitis. At the same time, the brain MRI showed T2 hyperintensity in the right thalamus (Figure 1H). Nine of seventeen patients underwent follow-up brain MRI during the remission phase. Apart from Patient 2, who developed a new lesion during the relapse, one patient (1/9, 11%, patient 3) had subclinical lesions. Patient 3 exhibited asymptomatic radiological activity on the second month of disease onset, characterized by T2 hyperintensity in the right globus pallidus, but did not receive immunotherapy. The MRI lesion absorption has disappeared after the fifth month of onset (Figures 1J–L). Additionally, seven patients (7/9, 78%) had normal brain MRI during follow-up examinations during the remission phase. All patients achieved complete clinical remission at the final follow-up, with MRS scores all at 0.

## 4 Discussion

In recent years, the disease spectrum of MOGAD has continually broadened, with studies uncovering rare and atypical demyelinating presentations, such as isolated seizures (4, 5), cortical encephalitis (5, 16), aseptic meningitis (3, 5), overlapping syndrome of MOGAD and anti-N-methyl-D aspartate receptor encephalitis (5) or Leukodystrophy-like phenotype (17). Despite several manuscripts offering diagnostic recommendations for MOGAD (18–20) and the International Panel on MOG-Antibody Associated Demyelinating Diseases proposing criteria in 2023 (1), these guidelines do not comprehensively cover all clinical phenotypes associated with MOGAD. Therefore, current criteria might miss these cases with normal MRI.

Encephalitic syndromes with MOG antibodies and normal brain MRI have been sporadically reported in previous reports (3, 6–8). In a comprehensive study focusing on “Investigating the 2023 MOGAD Criteria in Children and Adults With MOG-Antibody Positivity Within and Outside Attacks,” three children also exhibited cerebral syndrome, pleocytosis, and normal brain MRI findings (with no radiological delay observed in repeat MRI studies conducted 1–2 weeks after the initial assessment) (7). In addition, radiologic lag has also been reported in the acute phase of MOGAD (8, 12). Therefore, current criteria might miss these cases with normal MRI or radiologic lag. This emerging phenotype of MOGAD presents with normal brain imaging, which contrasts with the typical demyelinating lesions observed in other forms of the disease and may lead to misdiagnosis. Consequently, heightened awareness and vigilance are crucial. Here, we summarized the clinical significance of MOG antibodies positive encephalitis with normal brain MRI for a better understanding of its diagnosis, treatment and prognosis.

The proportion of encephalitic syndromes associated with MOG antibodies positive and normal brain MRI findings in our center was relatively low, accounting for 4.67% of all MOGAD cases (17/364). In a previous study, MOGAD with normal brain MRI represented only 2% of the 133 pediatric cases (none was identified among 124 adults) (7). These patients tended to have an older onset age, with an average of 9.2 ± 2.8 years, and the majority (15/17, 89%) had their onset after 7 years of age. This is slightly older than the median age of 6.2 years reported in previous study for pediatric demyelinating and encephalitic syndromes associated with MOG antibodies (6). In our study, the main clinical manifestations of the patients were fever (17/17, 100%), headaches (17/17, 100%), slightly altered mental status (17/17, 100%), seizures (6/17, 35%; one with status epilepticus), vomiting (6/17, 35%), decreased binocular vision (2/17, 12%) and hemiplegia (1/17, 6%). Interestingly, fever was a prominent symptom, with a median duration of 15 days (IQR: 7.5–21 days) and 13 patients experiencing fever for more than a week (range: 10–30 days) before initiating immunotherapy. Additionally, these patients presented with relatively mild altered mental status, such as lethargy or somnolence, and their clinical manifestations were largely similar to those reported in cases of aseptic encephalitis (3). Compared to non-ADEM encephalitis, the symptoms in our patients were relatively milder (6). A previous study by Armangue et al. reported the clinical features of 22 patients with non-ADEM encephalitis, including decreased level of consciousness (22/22, 100%), seizures (14/22, 64%; 10 with status epilepticus), fever (13/22, 59%), abnormal behavior (11/22, 50%), motor deficits (9/22, 40%), abnormal movements (8/22, 36%), and brainstem cerebellar dysfunction (5/22, 23%) (6). However, only two of these 22 patients had normal brain MRI, while the remaining 20 exhibited imaging changes inconsistent with ADEM (6).

In our study, the blood white blood cell (WBC) count was 20.63 ± 7.09 × 109/L, with the majority of cases (15/17, 88%) exhibiting leukocytosis and elevated neutrophil ratios in all patients. However, the majority of patients (13/17, 76%) exhibited normal levels of PCT and CRP, with only a minority experiencing slight elevations. All patients presented with CSF leukocytosis (median: 82/mm^3^, IQR: 49–155/mm^3^), along with normal glucose levels in the CSF. Additionally, a small subset of patients (6/17, 35%) had mild elevated CSF protein levels. MOG antibody-positive encephalitic syndromes, when accompanied by normal brain MRI findings, can pose a diagnostic challenge due to their similarity to infectious diseases. Nevertheless, the peripheral inflammatory changes and CSF abnormalities observed in these patients differ from the typical patterns seen in bacterial meningitis and viral encephalitis. Furthermore, tests for herpes simplex virus, Epstein–Barr virus, *Mycoplasma pneumoniae*, and bacteria in the CSF were all negative, and next-generation sequencing of CSF from 10 patients also yielded negative results. Importantly, all patients failed to show clinical improvement after receiving antibiotics or antiviral therapy. Based on these test results and the patients’ response to treatment, we were able to reasonably exclude the diagnosis of infectious encephalitis.

All of the patients had a good response to first-line immunotherapy, further supporting the diagnosis of MOGAD. This good response to immunotherapy during the acute phase aligns with the majority of other MOGAD phenotypes reported in previous studies (6, 21, 22). Following a median follow-up duration of 17 months (IQR: 6.5–40 months), all patients achieved full recovery, with only one patient (1/17, 6%) experiencing a relapse. A large mixed adult and pediatric cohort with 252 MOG antibodies positive patients showed that 78% of these patients had a good (not further specified) or full recovery from the initial attack, with more often a full recovery in pediatric patients and patients presenting with ON and ADEM/ADEM-like phenotypes (22). After a median follow-up of 42 months (IQR: 22–67 months), 33 (28%) of the 116 patients with demyelinating and encephalitic syndromes with MOG antibodies positive had relapses, including 17 (17%) of 100 diagnosed at first episode (6). Gu et al. reported 17 patients with MOG antibodies positive aseptic meningitis-like attack, among which 4 cases relapsed. Among the relapsing 4 patients, 7 relapses occurred in total, including 3 attacks of ADEM-like, 2 attacks of cortical encephalitis, 1 attack of meningitis and 1 attack of isolated-ON (3). The recurrence rate observed in our study differs from that reported by Gu et al., potentially due to our exclusive inclusion of patients with normal brain MRI findings, whereas the case series reported by Gu et al. partially involved demyelinating lesions and meningeal enhancement. Compared to other phenotypes of MOGAD, the encephalitis phenotype accompanied by normal brain MRI appears to have a more favorable prognosis.

In our study, we observed that only one patient (patient 3) exhibited asymptomatic radiological activity 2 months after onset, characterized by a T2 hyperintense lesion in the right pallidum, which did not show contrast enhancement on T1-weighted imaging. The MRI lesion absorption has disappeared after the fifth month of onset without immunotherapy (Figures 1J–L). During the 38-month follow-up period, patient 3 did not receive chronic immunotherapy and did not experience any clinical recurrences. New brain remission lesions were reported in 10 of 74 children with MOGAD, most commonly within the first month post-onset, with a positive predictive value for clinically relapsing disease of only 20% (23). Therefore, detection of asymptomatic lesions alone need not prompt initiation of chronic immunotherapy (23).

It is noteworthy that patient 15 and patient 16 without receiving immunotherapy developed bilateral optic neuritis on the 20th and 28th day of the disease course, respectively. Among the 17 reported cases of MOGAD with aseptic meningitis-like attacks, 5 patients had new neurological manifestations, two of visual deterioration, two of ataxia, and one of abnormal mental behavior (3). In previous studies, it was found that early treatment of the first acute MOGAD attack is associated with a reduction in the proportion of recurrent courses and an increased likelihood of conversion to seronegative for MOG-IgG (24, 25). Thus, it is worthwhile to investigate whether prompt immunotherapy can prevent the emergence of new neurological manifestations.

The pathological mechanism of MOG antibodies positive encephalitis with normal brain MRI imaging is unknown. MOG is formed by oligodendrocyte plasma membranes that are wrapped around neuronal axons in the CNS. MOG is a protein expressed by oligodendrocytes and localized to the outermost lamellae of myelin (26). Therefore, MOGAD should be just associated with inflammatory demyelinating diseases of white matters in nervous system. However, in previous reports, a minority of MOGAD patients exhibit normal imaging or non-demyelinating lesions, such as isolated seizures (4) and cortical encephalitis (16). For instance, the patients with isolated seizures may present with unremarkable brain MRI findings at onset, but subsequently develop typical demyelinating events with MRI abnormalities during follow-up, accompanied by persistent MOG antibodies (4). The interval between isolated seizures and a new demyelinating event can range from months to years, suggesting an underlying immunological pathogenesis that may already be present at the onset of seizures (4). Furthermore, patients with MOG antibodies positive cortical encephalitis have shown mild inflammatory changes in the cortex and subcortex without distinct demyelination (27, 28). Therefore, some MOGAD cases do not exhibit demyelinating changes on imaging during the acute phase and may even appear normal radiographically. This could be due to the lack of a direct pathogenic relationship with MOG antibodies or the possibility that some changes are not detectable by conventional MRI. Advanced imaging techniques such as 7.0 T ultrahigh-field MRI or Positron Emission Tomography-MRI may uncover some potential changes, which requires further research and validation. In 8 out of 33 (24%) pediatric cases of MOG-antibody-associated encephalitis, initial MRI scans of the brain were normal (8). However, subsequent imaging during attacks revealed abnormalities in 4 of these children (8). Although our 15 patients underwent a follow-up brain MRI during the acute phase, the possibility of radiologic lag cannot be ruled out. While the pathogenesis of MOG antibodies in encephalitis with normal brain MRI remains unclear, we propose that this type may be regarded as a new spectrum associated with MOG antibodies. As the spectrum of MOGAD continues to expand, the diagnostic criteria for MOGAD require validation and further optimization.

We acknowledge several limitations of this study. First, not all patients underwent spinal MRI to determine the presence of subclinical spinal lesions. Secondly, two children did not undergo a follow-up brain MRI during the acute phase, so it is impossible to completely rule out the possibility of new radiological lesions developing. Third, it cannot be guaranteed that all patients underwent MRI scans using the same machine. Fourth, not all patients undergo CNS next-generation sequencing. Fifth, the follow-up time was relatively short for some patients. Finally, although this is the largest case series known to us currently, the number of cases is still small. More prospective multicenter studies with larger samples are needed to further explore the clinical characteristics, treatment, and prognosis of this type.

## 5 Conclusion

We recommend that patients with clinical manifestations of encephalitis, even if the brain MRI are normal, should undergo early testing for anti-MOG antibody if they exhibit the following characteristics: (1) persistent fever; (2) elevated peripheral blood WBC counts, while PCT and CRP are normal or slightly elevated; (3) mild elevation of CSF WBC counts, normal or mild elevated protein, and normal cerebrospinal fluid glucose; (4) ineffective antibiotics or antiviral therapy. Encephalitis with normal brain MRI may be regarded as a potential new spectrum associated with MOG antibodies, meriting additional exploration and consideration.

## Data Availability

The original contributions presented in the study are included in the article/supplementary material, further inquiries can be directed to the corresponding authors.
